# Sex-Related Differences in Non–Pulmonary Vein Triggers During Initial Atrial Fibrillation Ablation

**DOI:** 10.1001/jamanetworkopen.2025.29527

**Published:** 2025-08-28

**Authors:** Corentin Chaumont, Alireza Oraii, Oriol Rodriguez-Queralto, Adrian Petzl, Erica Zado, Balaram Krishna J. Hanumanthu, Timothy M. Markman, Matthew C. Hyman, Cory M. Tschabrunn, Andres Enriquez, Poojita Shivamurthy, Ramanan Kumareswaran, Michael P. Riley, David Lin, Robert D. Schaller, Saman Nazarian, David J. Callans, Gregory E. Supple, Fermin C. Garcia, David S. Frankel, Sanjay Dixit, Frederic Anselme, Francis E. Marchlinski

**Affiliations:** 1 Section of Cardiac Electrophysiology, Division of Cardiovascular Medicine, Department of Medicine, Hospital of the University of Pennsylvania, Philadelphia; 2 CHU Rouen, Department of Cardiology, Univ Rouen Normandie, Inserm EnVI UMR 1096, Rouen, France

## Abstract

**Importance:**

Atrial fibrillation (AF) ablation aims to eliminate all AF triggers. Non–pulmonary vein (PV) triggers are observed in up to 10% of patients referred for a first AF ablation procedure. However, there are limited data on sex-specific differences in non-PV triggers.

**Objective:**

To assess the sex-specific prevalence, type, and site of origin of non-PV triggers among patients referred for a first-time AF ablation procedure.

**Design, Setting, and Participants:**

This cohort study was conducted at the Hospital of the University of Pennsylvania among 2038 consecutive patients who underwent first-time AF ablation between January 1, 2018, and December 31, 2022, and received at least 1 step of the non-PV trigger provocation protocol. Statistical analysis was performed from October 2024 to May 2025.

**Exposures:**

Patients underwent a first-time AF ablation and received at least 1 step of the non-PV trigger provocation protocol: (1) cardioversion of spontaneous AF to identify any spontaneous triggers during sinus rhythm, (2) isoproterenol infusion up to 20 to 30 µg/min, and (3) rapid atrial burst pacing. Non-PV triggers were defined as non-PV ectopic beats triggering AF or sustained focal atrial tachycardia (AT).

**Main Outcomes and Measures:**

The primary end point was atrial arrhythmia recurrence within 1 year, defined as any atrial arrhythmia lasting more than 30 seconds after the 90-day blanking period.

**Results:**

A total of 2038 patients were included (mean [SD] age, 64.7 [10.5] years; 1369 men [67.2%]). The prevalence of non-PV triggers was significantly higher among women than men (72 of 669 [10.8%] vs 91 of 1369 [6.6%]; *P* = .001). Right atrial triggers were more common among women than men (42 of 669 [6.3%] vs 44 of 1369 [3.2%]; *P* = .001). Left atrial triggers were not significantly different among women compared with men (35 of 669 [5.2%] vs 50 of 1369 [3.7%]; *P* = .09). Although the proportion of non-PV triggers initiating AF and sustained AT was balanced among women (AF, 34 of 72 [47.2%]; AT, 36 of 72 [50.0%]; and both, 2 of 72 [2.8%]), non-PV triggers initiating AT were predominant among men (AT, 62 of 91 [68.1%]; AF, 28 of 91 [30.8%]; and both, 1 of 91 [1.1%]). Triggers from 2 or more locations were more frequent among women than men (18 of 72 [23.6%] vs 11 of 91 [12.1%]; *P* = .03). Among patients for whom non-PV triggers were identified (n = 163), women had a higher 1-year atrial arrhythmia recurrence rate (adjusted hazard ratio, 1.77 [95% CI, 1.02-3.08]; *P* = .04).

**Conclusions and Relevance:**

In this cohort study of 2038 patients, women exhibited a higher prevalence of non-PV triggers, especially from the right atrium, than men. Women with non-PV triggers were more likely to have multiple triggers and worse postablation outcomes. These findings highlight the importance of performing systematic provocation protocol with increased vigilance among women, particularly for those with additional risk factors.

## Key Points

**Question:**

What are the sex-specific differences in prevalence, type, and site of origin of non–pulmonary vein (PV) triggers among patients undergoing first-time ablation for atrial fibrillation (AF)?

**Findings:**

In this cohort study of 2038 patients, the prevalence of non-PV triggers was higher among women than men (10.8% vs 6.6%), largely owing to a higher rate of right atrial foci. Women with non-PV triggers were more likely to have multiple triggers and worse postablation outcomes.

**Meaning:**

This study suggests that sex is significantly associated with the prevalence and characteristics of non-PV triggers identified during AF ablation, highlighting the need for a personalized approach to provoking, localizing, and ablating AF triggers among women and men.

## Teaser

This cohort study assesses the sex-specific differences in prevalence, type, and site of origin of non–pulmonary vein triggers among patients undergoing first-time ablation for atrial fibrillation.

## Introduction

The primary goal of atrial fibrillation (AF) catheter ablation is the elimination of all arrhythmogenic foci that trigger or sustain AF.^1,2^ Although the pulmonary veins (PVs) are well established as the main source of AF triggers,^3,4^ increasing evidence suggests the important role of additional triggers from non-PV locations.^5,6,7,8^ If not identified and ablated, these non-PV triggers have been shown to increase the risk of AF recurrence.^5,9,10^ Despite improvements in the efficiency and safety of PV isolation (PVI),^11^ AF recurrence rates after ablation have remained relatively unchanged over the past decade.^12^ Given the increasing prevalence of AF, there is an increasing need for new, tailored ablation approaches beyond PVI. Previous research has developed a preprocedural screening tool for first-time ablation procedures to identify patients who are at higher risk of non-PV triggers.^13^ Female sex was associated with a nearly 2-fold increase in the risk of non-PV triggers. However, data on sex-specific differences in non-PV triggers remain limited. Exploring these differences is crucial for advancing our knowledge of AF pathophysiology and for providing more personalized non-PV trigger testing and ablation strategies. In this study, we aimed to analyze the prevalence, location, and characteristics of non-PV triggers based on sex and to refine the sex-specific risk factors of non-PV triggers. In addition, we aimed to evaluate whether sex is associated with outcomes among patients for whom non-PV triggers are identified and ablated.

## Methods

### Study Design

Consecutive patients who underwent first-time AF catheter ablation between January 1, 2018, and December 31, 2022, were identified from the AF ablation registry at the Hospital of the University of Pennsylvania. This registry is designed to prospectively collect demographic information, baseline risk factors, echocardiographic findings, and intraprocedural details of patients undergoing AF ablation at the Hospital of the University of Pennsylvania. Patients were excluded if they had a history of heart or lung transplant or did not undergo non-PV trigger provocative maneuvers. All patients provided written informed consent, and the study received approval from the Hospital of the University of Pennsylvania institutional review board, in accordance with the guidelines of the Declaration of Helsinki.^14^ This study adhered to the Strengthening the Reporting of Observational Studies in Epidemiology (STROBE) reporting guideline for cohort studies.

### Definition of Non-PV Triggers

Non-PV triggers were defined as ectopic beats originating from outside the PVs that triggered AF or sustained focal atrial tachycardia (AT). Atrioventricular nodal reentrant tachycardia (AVNRT), atrioventricular reciprocating tachycardia (AVRT), macro-reentrant atrial flutters, and isolated frequent premature atrial beats that did not trigger AF were not considered non-PV triggers.

### Provocation and Mapping Protocol

The non-PV triggers provocation and mapping protocol has been previously described.^15^ In brief, ablation procedures were performed under general anesthesia with jet ventilation. An irrigated contact-force radiofrequency ablation catheter (Thermocool SmartTouch, Biosense Webster Inc or TactiCath, Abbot Inc) and a multielectrode mapping catheter were advanced into the left atrium (LA). Two decapolar catheters were placed in the coronary sinus and along the crista terminalis, with the distal electrode extending into the superior vena cava. All patients underwent bilateral wide-circumferential PVI with irrigated contact-force radiofrequency ablation catheters. After confirmation of PVI with entrance and exit block, pharmacologic challenges and pacing maneuvers were performed to identify non-PV triggers. The provocation protocol consisted of the following steps: (1) electrical cardioversion of spontaneous AF to identify triggers during sinus rhythm; (2) incremental isoproterenol infusion starting at 3 µg/min, with increases every 3 to 5 minutes to 6, 12, and 20 to 30 µg/min; and (3) rapid atrial burst pacing, during isoproterenol washout, starting at a cycle length of 250 milliseconds, with decrements of 10 milliseconds down to 180 milliseconds or until 2:1 atrial capture. Activation patterns recorded from the decapolar catheters in the coronary sinus and crista terminalis were used to regionalize the non-PV trigger site of origin. The multipolar mapping and ablation catheters were then repositioned to the suspected area for detailed mapping and localization. Identified sites of origin or the nearest anatomical structure where non-PV triggers commonly occur were then targeted with radiofrequency and the provocation maneuvers were repeated to search for additional triggers, if initiated. Non-PV triggers originating from the LA posterior wall were targeted with posterior wall isolation, and non-PV triggers originating from the superior vena cava were targeted with superior vena cava isolation. Additional linear lesions were performed when macro-reentrant atrial flutters were documented.

### Follow-Up

Patients with identified non-PV triggers were followed up through outpatient ambulatory cardiac (14-30 days) electrocardiographic monitoring; interrogation of insertable cardiac monitors, pacemakers, and implantable cardioverter-defibrillators; and office visits at 6 to 8 weeks, 6 months, and 1 year after the procedure. The primary end point was atrial arrhythmia recurrence within 1 year, defined as any atrial arrhythmia lasting more than 30 seconds after the 90-day blanking period.

### Statistical Analysis

Statistical analysis was performed from October 2024 to May 2025. Patients were stratified by sex. Continuous variables were reported as mean (SD) values, and categorical variables were expressed as counts and percentages. The *t* test was used to compare continuous variables, while the χ^2^ test was applied to assess differences in proportions. Logistic regression models were used separately for male and female groups to evaluate the association between preprocedural factors and the identification of non-PV triggers during the procedure. Results were reported as odds ratios (ORs) with 95% CIs. All factors with a univariable *P* < .30 were entered into a multivariable model.

Among patients with non-PV triggers, 1-year atrial arrhythmia–free survival was plotted using the Kaplan-Meier method. Cox proportional hazards regression was performed to assess the association of sex with postablation outcomes, after adjusting for age, persistent AF, hypertension, diabetes, heart failure with reduced ejection fraction (HFrEF), and moderate-to-severe LA dilatation (defined as an LA volume index ≥42 mL/m^2^, an LA diameter ≥4.7 cm in men, or an LA diameter ≥4.3 cm in women detected by echocardiography).^16,17^ All *P* values were from 2-sided tests and results were deemed statistically significant at *P* < .05.

## Results

A total of 2038 patients (mean [SD] age, 64.7 [10.5] years; 1369 men [67.2%] and 669 women [32.8%]) who underwent first-time AF catheter ablation and received at least 1 step of the non-PV trigger provocation protocol were included in the study (eFigure 1 in Supplement 1). Baseline characteristics by sex are described in Table 1. Provocation maneuvers were similarly distributed between the 2 groups. Specifically, 548 men (40.0%) and 251 women (37.5%) underwent cardioversion of spontaneous AF (*P* = .30), 1277 men (93.3%) and 634 women (94.8%) received isoproterenol infusion (*P* = .23), and 1020 men (74.5%) and 485 women (72.5%) underwent atrial burst pacing (*P* = .36). The mean (SD) maximum dose of isoproterenol administered during the provocation protocol was comparable between males and females (17 [5.5] µg/min and 16.9 [5.6] µg/min, respectively; *P* = .75).

**Table 1.** Baseline Characteristics According to Sex^a^

Characteristic	No. (%)	
	Women (n = 669)	Men (n = 1369)
Age, mean (SD), y	66.8 (10.6)	63.6 (10.2)
Persistent AF	241 (36.0)	653 (47.7)
BMI, mean (SD)	29.7 (6.9)	29.9 (5.4)
Hypertension	442 (66.1)	933 (68.2)
Diabetes	94 (14.1)	261 (19.1)
Obstructive sleep apnea	166 (24.8)	455 (33.2)
CVA or TIA	57 (8.5)	107 (7.8)
HFrEF	40 (6.0)	213 (15.6)
Hypertrophic cardiomyopathy	29 (4.3)	39 (2.8)
Coronary artery disease	39 (5.8)	232 (16.9)
Cardiac sarcoidosis or amyloidosis	3 (0.4)	23 (1.7)
Chronic lung disease	60 (9.0)	75 (5.5)
Chronic kidney disease	29 (4.3)	104 (7.6)
Sinus node dysfunction	51 (7.6)	61 (4.5)
Moderate or severe LA enlargement^b^	180 (26.9)	387 (28.3)
Cardiac surgery	52 (7.8)	136 (9.9)

Abbreviations: AF, atrial fibrillation; BMI, body mass index (calculated as weight in kilograms divided by height in meters squared); CVA, cerebrovascular accident; HFrEF, heart failure with reduced ejection fraction; LA, left atrium; TIA, transient ischemic attack.

^a^ There were no missing data for baseline characteristics.

^b^ Moderate or severe LA enlargement was defined as LA volume index of 42 mL/m^2^ or greater, LA diameter of 4.7 cm or greater in men, or LA diameter of 4.3 cm or greater in women on echocardiography.

### Prevalence and Characteristics of Non-PV Triggers According to Sex

**Figure 1.**
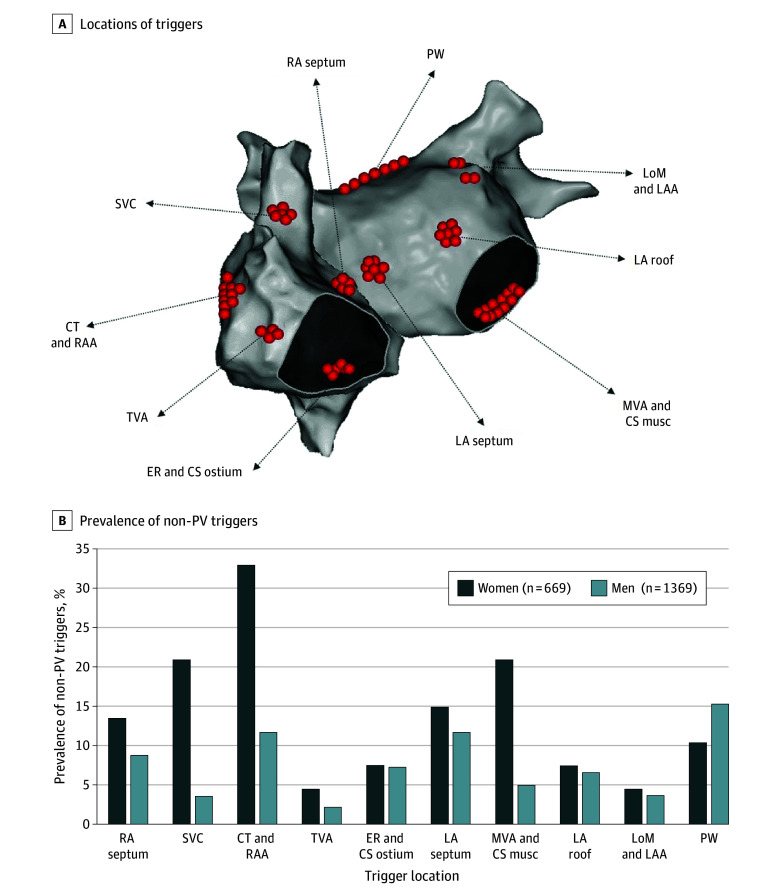
Frequency of Non–Pulmonary Vein (PV) Triggers, Stratified by Sex and Location A, Locations of 201 triggers in 163 patients (91 men and 72 women). B, Prevalence of triggers per 1000 patients. CS, indicates coronary sinus; CT, crista terminalis; ER, eustachian ridge; LAA, left atrial appendage; LA, left atrium; LoM, ligament of Marshall; musc, musculature; MVA, mitral valve annulus; PW, posterior wall; RAA, right atrial appendage; RA, right atrium; SVC, superior vena cava; and TVA, tricuspid valve annulus.

Non-PV triggers were significantly more frequent among women than men, with 10.8% of women (72 of 669) exhibiting non-PV triggers compared with 6.6% of men (91 of 1369) (*P* = .001). After adjusting for age, persistent AF, hypertension, diabetes, HFrEF, and moderate-to-severe LA dilatation, female sex remained associated with an increased risk of non-PV triggers (adjusted OR, 1.81 [95% CI, 1.28-2.56]; *P* < .001). The distribution and location of non-PV triggers by sex are shown in Figure 1. Right atrial triggers were more common among women than men (6.3% [42 of 669] vs 3.2% [44 of 1369]; *P* = .001). The prevalence of LA triggers among women compared with men was not significantly different (5.2% [35 of 669] vs 3.7% [50 of 1369]; *P* = .09). Among the 163 patients with non-PV triggers, triggers from 2 or more locations were more frequently observed among women than men (23.6% [18 of 72] vs 12.1% [11 of 91]; *P* = .03).

The prevalence of triggers from the superior vena cava (2.1% [14 of 669] vs 0.4% [5 of 1369]), crista terminalis (3.3% [22 of 669] vs 1.2% [16 of 1369]), and mitral valve annulus or coronary sinus musculature (2.1% [14 of 669] vs 0.5% [7 of 1369]) was 3 to 6 times higher among women than men (Figure 1). Although the proportion of non-PV triggers initiating AF and sustained AT was balanced among women (AT, 36 of 72 [50.0%]; AF, 34 of 72 [47.2%]; and both, 2 of 72 [2.8%]), triggers initiating AT (62 of 91 [68.1%]) were predominant compared with those initiating AF among men (28 of 91 [30.8%] and 1 of 91 patients with both [1.1%]) among men.

### Characteristics of Patients With Non-PV Triggers Across Sex Categories

Women with non-PV triggers were significantly older than men (69.1 [8.6] vs 65.8 [10.5] years; *P* = .03), but generally had fewer comorbidities (eTable 1 in Supplement 1). Compared with men, women had a lower prevalence of coronary artery disease (6.9% [5 of 72] vs 28.6% [26 of 91]; *P* < .001), chronic kidney disease (2.8% [2 of 72] vs 14.3% [13 of 91]; *P* = .01), and history of coronary artery bypass grafting (2.8% [2 of 72] vs 12.1% [11 of 91]; *P* = .03). Heart failure with reduced ejection fraction was also significantly more common among men than women with non-PV triggers (31.9% [29 of 91] vs 5.6% [4 of 72]; *P* < .001).

### Risk Factors of Non-PV Triggers Across Sex Categories

The univariable and multivariable associations between baseline risk factors and the presence of non-PV triggers are reported in Table 2 for men and Table 3 for women. In the multivariable model, cardiac sarcoidosis or amyloidosis and moderate or severe LA enlargement were identified as significant risk factors associated with non-PV triggers in both sex categories. A body mass index (BMI [calculated as weight in kilograms divided by height in meters squared]) less than 25 was significantly associated with non-PV triggers among women (multivariable OR, 1.76 [95% CI, 1.02-3.02]; *P* = .04; Table 3), but this association was not observed among men (univariable OR, 0.99 [95% CI, 0.56-1.75]; *P* = .96; Table 2). Conversely, HFrEF was a significant risk factor of non-PV triggers among men (multivariable OR, 1.73 [95% CI, 1.02-2.94]; *P* = .04; Table 2) but not among women (univariable OR, 0.92 [95% CI, 0.32-2.65]; *P* = .87; Table 3).

**Table 2.** Association Between Preprocedural Risk Factors and Non-PV Triggers at First-Time Atrial Fibrillation Ablation Among Men

Characteristic	Men (n = 1369)			
	Univariable OR	*P* value	Multivariable OR	*P* value
Age, y	1.03 (1.00-1.05)	.04	1.00 (0.98-1.03)	.89
Persistent AF	1.43 (0.93-2.20)	.10	0.86 (0.54-1.38)	.53
BMI <25	0.99 (0.56-1.75)	.96	NA	NA
Hypertension	1.60 (0.97-2.65)	.07	1.06 (0.61-1.85)	.84
Diabetes	1.39 (0.84-2.29)	.20	1.09 (0.62-1.90)	.77
Obstructive sleep apnea	1.55 (1.01-2.39)	.05	1.49 (0.94-2.38)	.09
CVA or TIA	1.32 (0.64-2.71)	.45	NA	NA
HFrEF	2.78 (1.74-4.44)	<.001	1.73 (1.02-2.94)	.04
Hypertrophic cardiomyopathy	1.18 (0.36-3.90)	.79	NA	NA
Coronary artery disease	2.08 (1.29-3.36)	.003	1.32 (0.74-2.35)	.34
Cardiac sarcoidosis or amyloidosis	6.6 (2.63-16.42)	<.001	5.21 (1.95-13.94)	.001
Chronic lung disease	1.24 (0.52-2.93)	.63	NA	NA
Chronic kidney disease	2.17 (1.16-4.06)	.02	1.06 (0.52-2.16)	.87
Sinus node dysfunction	2.59 (1.23-5.44)	.01	2.08 (0.92-4.70)	.08
Moderate or severe LA enlargement^a^	4.35 (2.81-6.75)	<.001	3.73 (2.31-6.04)	<.001
Cardiac surgery	2.42 (1.40-4.20)	.002	1.60 (0.85-3.00)	.15

Abbreviations: AF, atrial fibrillation; BMI, body mass index (calculated as weight in kilograms divided by height in meters squared); CVA, cerebrovascular accident; HFrEF, heart failure with reduced ejection fraction; LA, left atrium; NA, not applicable; OR, odds ratio; PV, pulmonary vein; TIA, transient ischemic attack.

^a^ Moderate or severe LA enlargement was defined as LA volume index of 42 mL/m^2^ or more, LA diameter of 4.7 cm or more in men, or LA diameter of 4.3 cm or more in women on echocardiography.

**Table 3.** Association Between Preprocedural Risk Factors and Non-PV Triggers at First-Time Atrial Fibrillation Ablation Among Women

Characteristic	Women (n = 669)			
	Univariable OR	*P* value	Multivariable OR	*P* value
Age, y	1.03 (1.00-1.05)	.05	1.01 (0.99-1.04)	.29
Persistent AF	1.80 (1.10-2.94)	.02	1.26 (0.73-2.16)	.41
BMI <25	1.57 (0.94-2.63)	.09	1.76 (1.02-3.02)	.04
Hypertension	0.89 (0.54-1.50)	.68	NA	NA
Diabetes	1.40 (0.74-2.67)	.30	NA	NA
Obstructive sleep apnea	1.01 (0.58-1.78)	.97	NA	NA
CVA or TIA	1.18 (0.51-2.71)	.70	NA	NA
HFrEF	0.92 (0.32-2.65)	.87	NA	NA
Hypertrophic cardiomyopathy	0.60 (0.14-2.59)	.50	NA	NA
Coronary artery disease	1.24 (0.47-3.27)	.67	NA	NA
Cardiac sarcoidosis or amyloidosis	17.0 (1.53-190.20)	.02	25.2 (2.04-313)	.01
Chronic lung disease	1.53 (0.72-3.25)	.27	1.26 (0.56-2.82)	.58
Chronic kidney disease	0.60 (0.14-2.59)	.50		
Sinus node dysfunction	1.61 (0.73-3.58)	.24	1.62 (0.70-3.74)	.26
Moderate or severe LA enlargement^a^	2.95 (1.79-4.86)	<.001	2.52 (1.45-4.38)	<.001
Cardiac surgery	3.15 (1.59-6.24)	<.001	2.83 (1.36-5.95)	.006

Abbreviations: AF, atrial fibrillation; BMI, body mass index (calculated as weight in kilograms divided by height in meters squared); CVA, cerebrovascular accident; HFrEF, heart failure with reduced ejection fraction; LA, left atrium; NA, not applicable; OR, odds ratio; PV, pulmonary vein; TIA, transient ischemic attack.

^a^ Moderate or severe LA enlargement was defined as LA volume index of 42 mL/m^2^ or more, LA diameter of 4.7 cm or more in men, or LA diameter of 4.3 cm or more in women on echocardiography.

### Association of Sex With Postablation Outcomes Among Patients With Non-PV Triggers

**Figure 2.**
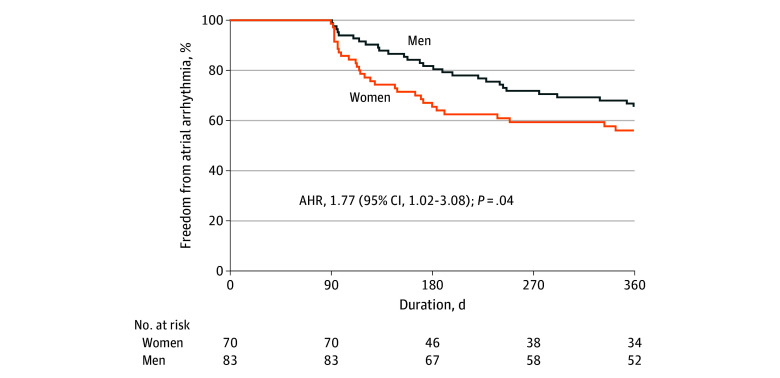
Freedom From Atrial Arrhythmia by Sex Among Patients for Whom Non–Pulmonary Vein Triggers Were Identified AHR indicates adjusted hazard ratio—adjusted for age, persistent atrial fibrillation, hypertension, diabetes, heart failure with reduced ejection fraction, and moderate-to-severe left atrium (LA) dilatation (defined as LA volume index ≥42 mL/m^2^, LA diameter ≥4.7 cm in men, or LA diameter ≥4.3 cm in women on echocardiography).

Among patients for whom non-PV triggers were identified, follow-up beyond the blanking period was available for 83 men and 70 women. Ten patients were lost to follow-up. Of these 153 patients, 52 (34.0%) underwent continuous rhythm monitoring with cardiac implantable electronic devices (eTable 2 in Supplement 1). Atrial arrhythmia recurrence within 1 year occurred among 28 of 83 men (33.7%) and 32 of 70 women (45.7%). After adjusting for age, persistent AF, hypertension, diabetes, HFrEF, and moderate or severe LA dilatation, female sex was associated with an increased risk of 1-year atrial arrhythmia recurrence (adjusted hazard ratio, 1.77 [95% CI, 1.02-3.08]; *P* = .04) (Figure 2; eTable 3 in Supplement 1).

## Discussion

To our knowledge, this is the first study to specifically examine sex-related differences in the characteristics of non-PV triggers and the outcomes after their ablation. The main findings are as follows: (1) women exhibited a higher prevalence of non-PV triggers compared with men, primarily due to right atrial non-PV triggers; (2) women were more likely to present with non-PV triggers originating from multiple locations; (3) non-PV triggers in women were equally distributed between AF and AT triggers, whereas in men, AT triggers predominated; and (4) although women with non-PV triggers had fewer comorbidities than their male counterparts, they had higher rates of AF recurrence after catheter ablation.

### Pathophysiological Hypothesis

Our data confirm a higher prevalence of non-PV triggers among women compared with men, aligning with previous findings.^18,19,20^ In a cohort of individuals undergoing first-time AF ablation, non-PV triggers were identified in 16.3% of women vs 6.9% of men.^19^ Another study including 431 patients reported a prevalence of 9.3% for non-PV triggers, with female sex significantly associated with an increased prevalence of right-sided non-PV triggers.^20^

Similar to the PVs, the extended myocardial sleeves encircling the SVC and coronary sinus are associated with AF triggers. The crista terminalis demarcates the transition between the primitive atrium and the sinus venosus. These transitional regions between nonexcitable, venous, or fibrous tissues and excitable myocardial tissue are known to frequently harbor ectopic foci. The regions where women exhibited a strong predominance of non-PV triggers compared with men (SVC, coronary sinus, and crista terminalis) share a common embryologic origin: the sinus venosus.^21^ In contrast, non-PV triggers were more frequent among men on the LA posterior wall, which has a different embryologic origin shared with the PVs. Meanwhile, triggers arising from the primitive atrium appeared to be equally distributed between sexes. A recent study exploring the embryologic origin of AF triggers (sinus venosus, primitive atrium, and common PV) highlighted that female sex and low BMI were specifically associated with non-PV triggers originating from the sinus venosus, aligning with our findings.^22^

The higher prevalence of non-PV triggers observed among women may be partly explained by a greater degree of atrial fibrosis, as reported in previous studies.^23^ This fibrotic remodeling, which appears to be more pronounced among women, may promote ectopic activity originating outside the PVs^20^ and is potentially associated with sex-related differences in AF pathophysiology. Although the underlying pathophysiological mechanisms are not fully understood, enhanced activation of the profibrotic transforming growth factor-β (TGF-β)/SMAD3 signaling pathway has been documented in women with AF. Specifically, increased expression of TGF-β1, TGF-β2, and collagen types I α2 and III α1 has been observed among women compared with men.^24^ The prevalence of sinus node dysfunction was significantly higher among women in our cohort, which may reflect a more advanced or diffuse atrial cardiomyopathy among women. This observation is consistent with prior findings suggesting that women with AF often exhibit more extensive atrial scarring despite a similar burden of cardiovascular risk factors.^25^

The difference in the prevalence and location of non-PV triggers between sexes may also be partly associated with hormonal influences, as estrogen and progesterone are known to play a role in arrhythmogenic mechanisms.^26^ Among premenopausal women, an increase in the number and duration of paroxysmal supraventricular tachycardia episodes was observed during the luteal phase, correlating with higher progesterone levels.^27^ Late onset of AF among women could be due to the reduced estrogen-mediated cardiovascular protection after menopause. However, postmenopausal hormone replacement therapy has so far shown contradictory results, with an increased risk of AF among patients receiving ongoing hormone replacement therapy.^28^

In addition, while little is known about the sex-related differences in the autonomic responses in the atria, the autonomic nervous system may be associated with these differences, given that women typically exhibit greater parasympathetic modulation.^29^ Vagal overactivity induces a shortening of the action potential and causes potent increases in heterogeneity of the atrial effective refractory period, creating substrate for arrhythmogenesis.^30^ Studying precisely the impact of these factors individually remains challenging. Further investigation is needed to elucidate the precise mechanisms by which vagosympathetic balance and hormonal fluctuations may precipitate non-PV triggers among women.

Finally, sex-related specificities in cellular electrophysiology may also be associated with the observed differences in non-PV triggers. Atrial fibrillation is associated with action potential remodeling, yet studies have not identified significant sex-related differences in key action potential characteristics—such as action potential duration, amplitude, or maximum upstroke velocity—when measured in right atrial trabeculae.^31^ To date, it remains unclear whether sex-related differences are present in the LA or whether ion channel expression differs between sexes across atrial regions. Atrial myocytes in women with AF have enhanced spontaneous Ca^2+^ release, with greater Ca^2+^ spark density compared with sinus rhythm and men with AF, favoring calcium waves that trigger a transient inward current and afterdepolarizations.^32^ This aberrant Ca^2+^ release in women likely summates as ectopic triggers in the form of delayed afterdepolarizations and thus points to a sex divergence in the mechanism of non-PV triggers.

### Risk Factors of Non-PV Triggers Across Sex Categories

Our study demonstrates that risk factors for non-PV triggers, which have been previously reported in the literature, are mostly common to both sexes, including LA dilation and infiltrative diseases. A previously described risk score can therefore be applied to both sexes.^13^ However, certain sex-specific risk factors may further facilitate risk stratification of patients for having non-PV triggers. A lower BMI appears to be a risk factor for non-PV triggers among women. Although this finding may seem surprising, it aligns with previous studies^10,33,34^ and may partly explain the U-shaped association between BMI and AF occurrence, with an increased risk with both low BMI (<18.5) and obesity.^35^ Conversely, heart failure seems to have a more pronounced association with presence of non-PV triggers among men in our cohort. Men with non-PV triggers presented with more comorbidities than women, suggesting that the underlying mechanisms of these triggers may differ between sexes.

Given the higher prevalence of non-PV triggers among women, a more systematic provocation protocol may be warranted for female patients, particularly for those with additional risk factors (including prior cardiac surgery, LA dilation, infiltrative cardiomyopathies, and low BMI). Therefore, a preprocedural risk assessment of patients based on these factors can identify those who are at greater risk of having non-PV triggers. Although current guidelines on AF catheter ablation do not differentiate recommended strategies by sex, our findings support the need for increased vigilance among women and suggest that sex-specific ablation strategies could be tested in future studies.

### Association of Sex With Long-Term Outcomes After AF Catheter Ablation

Previous studies have shown mixed results regarding outcomes of AF ablation between men and women. In the FIRE AND ICE (Cryoballoon or Radiofrequency Ablation for Paroxysmal Atrial Fibrillation) study, female sex was associated with a 37% higher risk of AF recurrence compared with male sex.^36^ In another study that aimed to develop a clinical scoring system to predict long-term freedom from AF after ablation, female sex was also identified as an independent predictor of recurrence.^37^ On the other hand, the CIRCA-DOSE (Cryoballoon vs. Irrigated Radiofrequency Catheter Ablation: Double Short vs. Standard Exposure Duration) substudy showed no sex-related difference in AF burden or 1-year recurrence after AF ablation.^38^ More recently, similar findings were reported with pulsed field ablation technology. In a retrospective cohort study using data from the MANIFEST-PF (Methods, Efficacy, and Safety on the Postapproval Clinical Use of Pulsed Field Ablation) registry, no significant differences in 1-year freedom from atrial arrhythmia were observed between male and female patients (79.0% vs 76.3%, respectively).^39^

Our study adds to the literature by focusing specifically on sex-related outcomes among patients with non-PV triggers. Although women with non-PV triggers had fewer comorbidities than men, they had a higher 1-year recurrence rate of atrial arrhythmia. This difference in midterm outcomes was observed despite using a similar stepwise provocation protocol and ablation approach. Although multiple non-PV triggers from different locations are more prevalent among women, which may complicate the identification and ablation of these triggers, it seems that other unidentified sex-related factors may also be associated with the observed differences in outcomes between sexes.

Despite a strict non-PV trigger provocation protocol and ablation, the recurrence rates (33.7% among men and 45.7% among women) may appear relatively high. As previously demonstrated, non-PV triggers are more frequently observed among patients with moderate-to-severe LA dilatation, sinus node dysfunction, prior cardiac surgery, and infiltrative cardiomyopathies.^13^ These conditions are also associated with a more abnormal atria, which may carry a poorer prognosis after AF ablation. Although non-PV triggers may represent markers of unhealthy atria, current expert consensus on catheter ablation of AF^40^ and the Heart Rhythm Society statement on catheter ablation of AF^41^ indicate that identifying and targeting non-PV triggers during AF ablation is reasonable.

### Limitations

This study has some limitations. This is a single-center study conducted at a high-volume tertiary care center. Therefore, the generalizability of our findings needs to be confirmed in future multicenter studies. When categorizing patients into 2 groups based on sex, the sample sizes might be underpowered to achieve statistical significance for some risk factors. We did not consider AVNRT or AVRT as non-PV triggers, as we believe their sex-related mechanisms and risk factors may differ from those of atrial foci triggering AF or sustained focal AT. However, compelling evidence suggests that AVNRT or AVRT can also act as non-PV triggers and are not uncommon during first-time ablation procedures.^42,43^ Continuous rhythm monitoring was not available for all patients due to the registry-based nature of our study, which may have resulted in potential underdetection of recurrent episodes. Finally, detailed information on atrial scarring was not consistently available across our cohort, as procedures were performed by multiple operators over an extended period.

## Conclusions

In this cohort study of 2038 patients, women exhibited a higher prevalence than men of non-PV triggers, largely owing to a higher rate of right atrial foci. Women were more likely to present with non-PV triggers from multiple locations. Among patients for whom non-PV triggers were identified, female sex was associated with a higher 1-year atrial arrhythmia recurrence rate. These findings highlight the importance of performing systematic provocation protocol with increased vigilance among women, particularly for those with additional risk factors.

## Notes

Supplement 1. **eFigure.** Flow Chart **eTable 1.** Baseline Characteristics According to Sex Among Patients With Non-PV Triggers **eTable 2.** Baseline Characteristics According to Follow-Up Modality **eTable 3.** Association Between Pre-Procedural Risk Factors and One-Year Atrial Arrhythmia Recurrence (Multivariable Cox Model)

Supplement 2.
Data Sharing Statement
