# Supplementary material for: Sex-Related Differences in Non–Pulmonary Vein Triggers During Initial Atrial Fibrillation Ablation
Source: JAMA Netw Open. 2025 Aug 28;8(8):e2529527. doi: 10.1001/jamanetworkopen.2025.29527 (PMC12395318; doi:10.1001/jamanetworkopen.2025.29527)
Supplement: Supplement 1. — eFigure. Flow Chart eTable 1. Baseline Characteristics According to Sex Among Patients With Non-PV Triggers eTable 2. Baseline Characteristics According to Follow-Up Modality eTable 3. Association Between Pre-Procedural Risk Factors and One-Year Atrial Arrhythmia Recurrence (Multivariable Cox Model) [file jamanetwopen-e2529527-s001.pdf]

Supplementary Online Content

Chaumont C, Oraii A, Rodriguez-Queralto O, et al. Sex-related differences in non–pulmonary vein triggers during initial atrial fibrillation ablation. *JAMA Netw Open.* 2025;8(8):e2529527. doi:10.1001/jamanetworkopen.2025.29527

eFigure. Flow Chart

eTable 1. Baseline Characteristics According to Sex Among Patients With Non-PV Triggers

eTable 2. Baseline Characteristics According to Follow-Up Modality

eTable 3. Association Between Pre-Procedural Risk Factors and One-Year Atrial Arrhythmia Recurrence (Multivariable Cox Model)

This supplementary material has been provided by the authors to give readers additional information about their work.

© 2025 Chaumont C et al. *JAMA Network Open.*

eFigure. Flow Chart

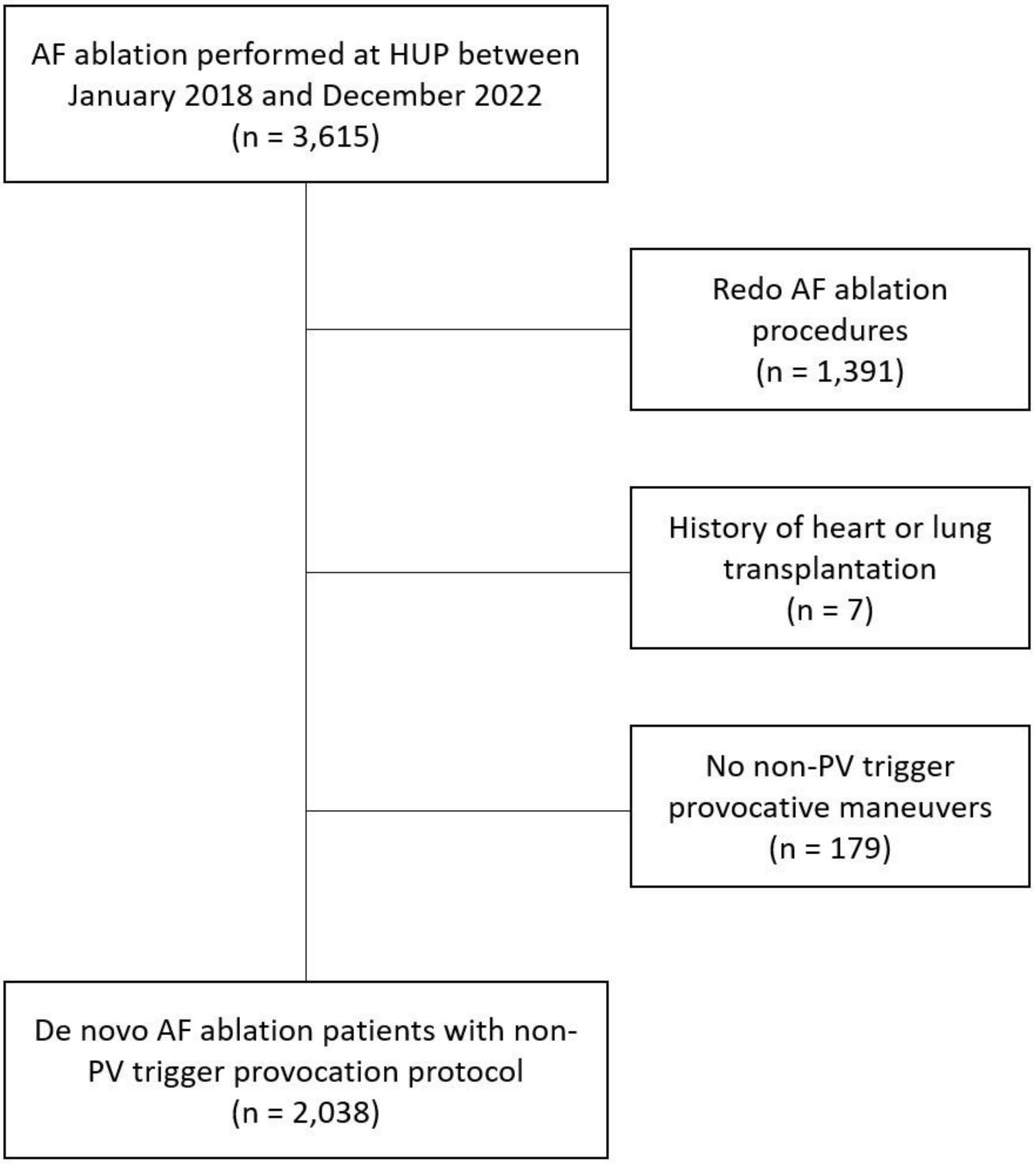

AF: atrial fibrillation; HUP: Hospital of the University of Pennsylvania; PV: pulmonary vein

© 2025 Chaumont C et al. *JAMA Network Open.*

eTable 1. Baseline Characteristics According to Sex Among Patients With Non-PV Triggers

	**Female (n = 72)**	**Male (n = 91)**	***p-value***
**Age, year**	69.1 ± 8.6	65.8 ± 10.5	0.03
**Persistent AF**	35 (48.6%)	51 (56%)	0.35
**BMI, kg/m^2**	28.3 ± 6.3	30.1 ± 5.8	0.07
**Hypertension**	46 (63.9%)	70 (76.9%)	0.07
**Diabetes**	13 (18.1%)	22 (24.2%)	0.35
**CVA / TIA**	7 (9.7%)	9 (9.9%)	0.97
**HFrEF**	4 (5.6%%)	29 (31.9%)	<.001
**Hypertrophic cardiomyopathy**	2 (2.8%)	3 (3.3%)	0.85
**Coronary artery disease**	5 (6.9%)	26 (28.6%)	<.001
**Cardiac Sarcoidosis/ amyloidosis**	2 (2.8%)	7 (7.7%)	0.17
**Chronic lung disease**	9 (12.5%)	6 (6.6%)	0.20
**Chronic kidney disease**	2 (2.8%)	13 (14.3%)	0.01
**Sinus node dysfunction**	8 (11.1%)	9 (9.9%)	0.8
**Moderate/severe LA enlargement[1]**	35 (48.6%)	55 (60.4%)	0.13
**Cardiac surgery**	13 (18.1%)	18 (19.8%)	0.78
**CABG**	2 (2.8%)	11 (12.1%)	0.03
**Valvular surgery**	9 (12.5%)	7 (7.7%)	0.31
**Congenital surgery**	4 (5.6%)	3 (3.3%)	0.48

Abbreviations: AF, atrial fibrillation; BMI, body mass index; CABG, coronary artery bypass graft surgery; CVA, cerebrovascular accident; HFrEF, heart failure with reduced ejection fraction; LA, left atrium; TIA, transient ischemic attack

[1]Moderate/severe LA enlargement was defined as LA volume index ≥42ml/m^2, LA diameter ≥4.7cm in men, or LA diameter ≥4.3cm in women on echocardiography.

© 2025 Chaumont C et al. *JAMA Network Open.*

eTable 2. Baseline Characteristics According to Follow-Up Modality

	**Continuous monitoring[1]** (n = 52)	**Standard follow-up** (n = 101)
**Female**	19 (36.5)	51 (50.5)
**Age, year**	66.7 10.5	67.3 9.5
**Persistent AF**	27 (51.9)	53 (52.5)
**BMI**	29.1 6.0	29.5 6.1
**Hypertension**	34 (65.4)	75 (74.3)
**Diabetes**	14 (26.9)	19 (18.8)
**Obstructive sleep apnea**	21 (40.4)	33 (32.7)
**CVA / TIA**	6 (11.5)	10 (9.9)
**HFrEF**	14 (26.9)	15 (14.9)
**Hypertrophic cardiomyopathy**	1 (1.9)	4 (4.0)
**Coronary artery disease**	15 (28.8)	12 (11.9)
**Cardiac Sarcoidosis/ amyloidosis**	5 (9.6)	4 (4.0)
**Chronic lung disease**	7 (13.5)	7 (6.9)
**Chronic kidney disease**	4 (7.7)	8 (7.9)
**Sinus node dysfunction**	11 (21.2)	5 (5.0)
**Moderate/severe LA enlargement[2]**	31 (59.6)	51 (50.5)
**Cardiac surgery**	17 (32.7)	13 (12.9)

Abbreviations: AF, atrial fibrillation; BMI, body mass index; CVA, cerebrovascular accident; HFrEF, heart failure with reduced ejection fraction; LA, left atrium; TIA, transient ischemic attack.

[1] Continuous monitoring included patients with a permanent pacemaker, an implantable cardioverter-defibrillator, or an implantable loop recorder.

[2] Moderate/severe LA enlargement was defined as LA volume index ≥42ml/m^2, LA diameter ≥4.7cm in men, or LA diameter ≥4.3cm in women on echocardiography.

© 2025 Chaumont C et al. *JAMA Network Open.*

eTable 3. Association Between Pre-Procedural Risk Factors and One-Year Atrial Arrhythmia Recurrence (Multivariable Cox Model)

	**Multivariable HR (95% CI)**	***p*-value**
**Age**	0.99 (0.96 – 1.02)	0.59
**Persistent AF**	1.19 (0.67 – 2.12)	0.55
**Hypertension**	1.03 (0.56 – 1.90)	0.93
**Diabetes**	0.89 (0.46 – 1.72)	0.72
**HFrEF**	1.05 (0.51 – 2.17)	0.89
**Moderate/severe LA dilatation[1]**	1.87 (1.04 – 3.36)	0.04
**Female sex**	1.77 (1.02 – 3.08)	0.04

[1]*Moderate/severe LA enlargement was defined as LA volume index ≥42ml/m2, LA diameter ≥4.7cm in men, or LA diameter ≥4.3cm in women on echocardiography.*

© 2025 Chaumont C et al. *JAMA Network Open.*
