# Supplementary material for: Sex-Related Differences in Non–Pulmonary Vein Triggers During Initial Atrial Fibrillation Ablation
Source: JAMA Netw Open. 2025 Aug 28;8(8):e2529527. doi: 10.1001/jamanetworkopen.2025.29527 (PMC12395318; doi:10.1001/jamanetworkopen.2025.29527)
Supplement: Supplement 2. — Data Sharing Statement [file jamanetwopen-e2529527-s002.pdf]

Data Sharing Statement

Chaumont. Sex-Related Differences in Non–Pulmonary Vein Triggers During Initial Atrial Fibrillation Ablation. *JAMA Netw Open*. Published August 28, 2025.
doi:10.1001/jamanetworkopen.2025.29527

Data
Data available: Yes
Data types: Deidentified participant data
How to access data: francis.marchlinski@pennmedicine.upenn.edu
When available: With publication

Supporting Documents
Document types: None

Additional Information
Who can access the data: Researchers whose proposed use of the data has been approved)
Types of analyses: For any purpose
Mechanisms of data availability: After approval of a proposal, without investigator support
